# The importance of contrast features in rat vision

**DOI:** 10.1038/s41598-023-27533-3

**Published:** 2023-01-10

**Authors:** Anna Elisabeth Schnell, Kasper Vinken, Hans Op de Beeck

**Affiliations:** 1 Department of Brain and Cognition and Leuven Brain Institute, KU Leuven, Leuven, Belgium; 2 Department of Ophthalmology, Children’s Hospital, Harvard Medical School, Boston, MA USA

**Keywords:** Psychology, Computational models

## Abstract

Models of object recognition have mostly focused upon the hierarchical processing of objects from local edges up to more complex shape features. An alternative strategy that might be involved in pattern recognition centres around coarse-level contrast features. In humans and monkeys, the use of such features is most documented in the domain of face perception. Given prior suggestions that, generally, rodents might rely upon contrast features for object recognition, we hypothesized that they would pick up the typical contrast features relevant for face detection. We trained rats in a face-nonface categorization task with stimuli previously used in computer vision and tested for generalization with new, unseen stimuli by including manipulations of the presence and strength of a range of contrast features previously identified to be relevant for face detection. Although overall generalization performance was low, it was significantly modulated by contrast features. A model taking into account the summed strength of contrast features predicted the variation in accuracy across stimuli. Finally, with deep neural networks, we further investigated and quantified the performance and representations of the animals. The findings suggest that rat behaviour in visual pattern recognition tasks is partially explained by contrast feature processing.

## Introduction

### Pattern vision

**Figure 1:**
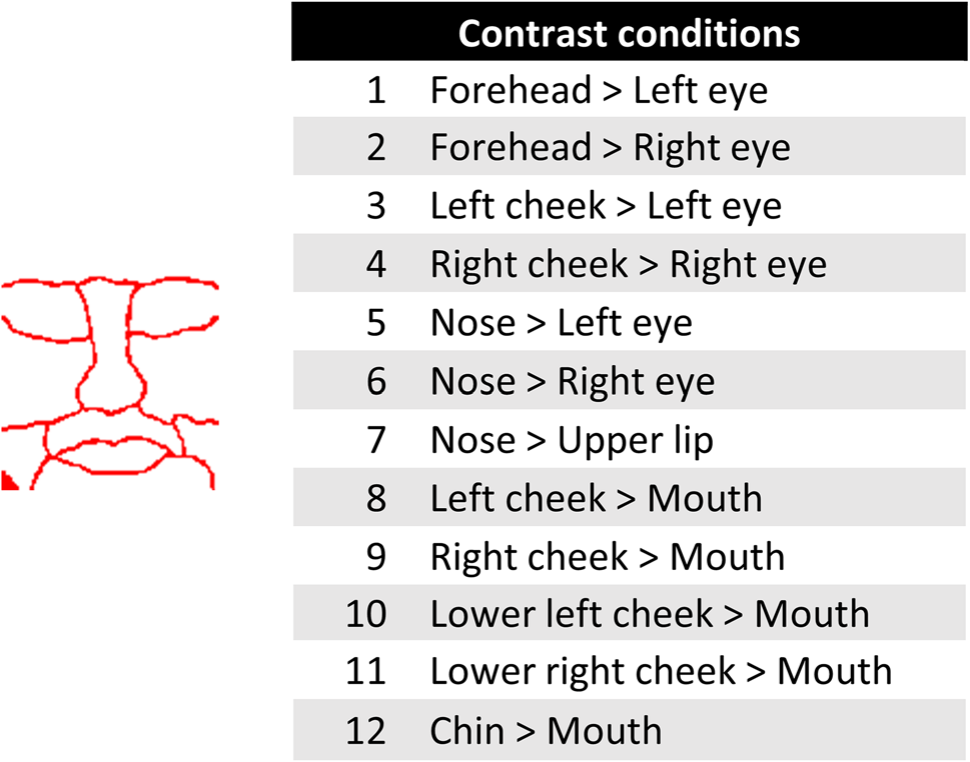
Template and twelve conditions of Sinha’s face detection model^5^. This template (red image on the left) is copied from Ohayon et al.^7^, their Fig. 6A. Based on this template, the twelve contrast conditions in the table on the right are created. This table is copied from Ohayon et al.^7^, their supplementary Fig. 4E.

All complex visual tasks such as object recognition and visual navigation start with an analysis of the spatial variation of light across the visual field, referred to as pattern vision. Pattern vision may seem like a trivial task for many people, but which information is necessary to perform this visual task? Previous research on pattern vision has focused mostly upon shape, starting with the analysis of edges, local curvature and orientation, and moving further into the processing of more complex shape features^1,2^. Some important frameworks in this field include the pure vision framework of Marr^3^ and the recognition-by-components theory of Biederman^4^. Models of object recognition mostly focus upon this type of processing. However, there is another set of processes that might be involved in pattern recognition. Contrasts have been shown to be an important element in shape perception, particularly in the context of face detection^5,6^. Sinha has create a simple computational model for face detection based on illumination-invariant contrast features. This model encompasses a face template that is largely invariant to illumination changes. In this model, a face is detected in an image if twelve conditions are met. These conditions (table in Fig. 1) are pairwise ordinal contrast relationships across facial regions that investigate the luminance difference across two regions of a face. Each one of these conditions tests whether contrast polarity is along the direction predicted from illumination invariance considerations. In some cases this model can break, for example if the face is strongly illuminated from below. Figure 1 shows the template on which these twelve contrast conditions are based. This contrast feature framework was further supported by neurophysiological research in monkeys. Ohayon and colleagues^7^ investigated whether contrast features, i.e., features based on these contrast cues, are a fundamental building block for face selectivity in the macaque inferotemporal cortex (IT). They found a contrast polarity preference in the macaque IT, which they see as a prediction from the computational face detection model of Sinha^5^.

### Rat vision

Given the differentiation between two types of pattern vision in humans, in terms of shape versus contrast features, we can question which of the two processes is involved when other species like rats perform object recognition tasks. This discussion has been lingering in the background in previous studies on rodent vision^8–12^. Some studies have argued in favour of invariant object recognition and shape perception^8,9^, while other studies were interpreted more in terms of mid-level features and templates that might be compatible with processing in terms of contrast features^10^. Compared to primate vision, where contrast features already play a significant role, this strategy might be even more prominent in rodents. Due to their low visual acuity, it might be more likely that rats use contrast features rather than shape properties that require high precision vision which rats do not have. With low visual acuity, the details contained in high spatial frequency information are lost, highlighting the contrast cues contained in the lower frequencies. Also in humans, the use of contrast features might become more prominent for the detection of objects in images with very few fine details and thus mainly low frequency information^13^.

Given that contrast feature models have mostly been used to model human and monkey visual responses in the domain of faces, and the most directly relevant computational models were also developed in the context of face detection, we decided to focus upon that domain and study to which extent contrast features would help explain rat vision in a face categorization task. While we have the general hypotheses that rats use contrast features in many domains, the exact contrast features will be task dependent. Given that the normal living situation of rats does not require the detection of human faces, we assume that the contrast features used in this task will be induced by our training procedure. For that reason, our stimulus design will make sure that the contrast features that have been shown to be relevant for human and monkey face categorization (see e.g.^7^) are also informative in our training stimulus set.

**Figure 2:**
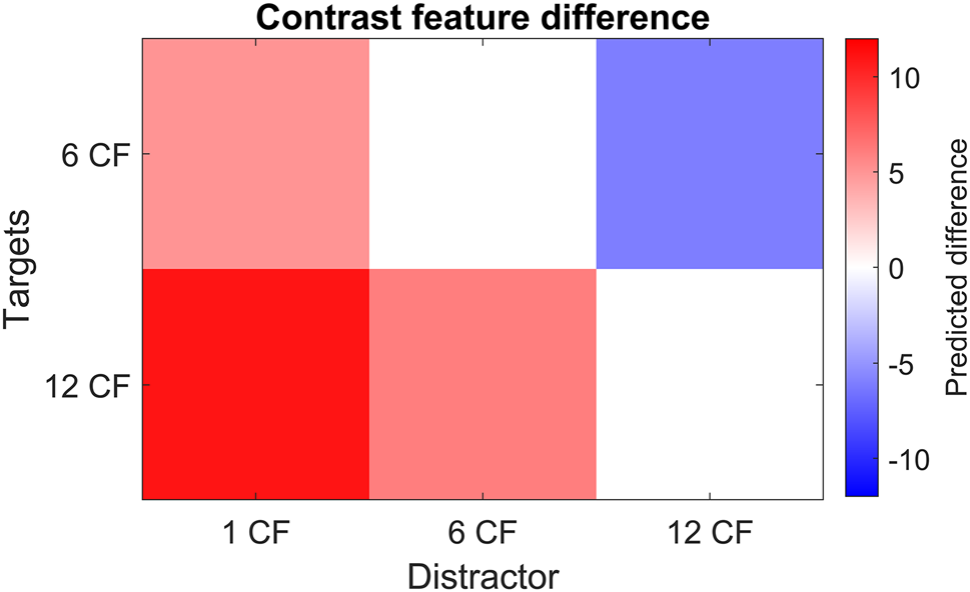
The simple contrast model. This matrix visualizes the simple contrast model that encompasses our six hypotheses. The x-axis represents distractors with 1, 6 and 12 correct contrast features, respectively. The y-axis represents the targets with 6 and 12 correct contrast features respectively. The colour scale indicates the difference in target and distractor contrast features. For positive values (red cells), we hypothesize that the higher the difference, the higher the performance of the animals, i.e. conditions 1, 2 and 4 in the table. For negative values, we expect the opposite (conditions 3, 5 and 6 in the table).

Previous research has shown that rats are capable of performing a face categorization task^14^. In this study, rats were successfully trained to classify faces from non-face objects and were able to generalize to new exemplars, even with modifications to the stimuli. However, we are unsure about the strategies that the animals used in this kind of task. In the current study, we investigate whether rats use contrast features to discriminate between faces and non-faces. Based on the list of the twelve contrast conditions (Fig. 1), faces and non-faces can have a different number of correct contrast features, depending on how many of these twelve conditions are met. Therefore, stimuli can be categorized depending on their number of correct features. In this study, rats were trained in a face categorization task where the faces (targets) and non-faces (distractors) have different number of correct contrast features. We have a total of six conditions hypothesizing the performance of the animals (Table 1). If the animals use contrast features in a face categorization task, as supposed by our contrast model, then we would expect the animals to perform well in the cases where the number of correct contrast features differ most between the target and distractor (see contrast model in Fig. 2).

**Table 1:** Our six hypotheses.

	Condition	T–D	Expected performance
1	12 CF (T) vs. 1 CF (D)	11	Very high
2	12 CF (T) vs. 6 CF (D)	6	High
3	12 CF (T) vs. 12 CF (D)	0	Chance level
4	6 CF (T) vs. 1 CF (D)	5	High
5	6 CF (T) vs. 6 CF (D)	0	Chance level
6	6 CF (T) vs. 12 CF (D)	-6	Lower than chance level

In the left and middle column of the table, T corresponds to Target and D corresponds to Distractor. The middle column represents the difference in contrast features between the target and the distractor. Each condition indicates a different number of correct contrast feature for the targets and the distractors.

### Computational modelling

If an effect of contrast features is found, then a further question is how we incorporate that strategy into the current mainstream computational thinking in vision sciences. Previous research has introduced deep convolutional neural networks (DNNs) as hierarchical model for ventral stream processing in primates^15,16^. In a recent study, these models were compared to rodent behavioural and neural data^17^. Vinken and Op de Beeck^17^ investigated the output of each layer of a neural network to see which layers contain stimulus representations that can support the same or better performance as rats in behavioural experiments. This allowed them to make an estimate of the level of vision processing required in rats performing object categorization tasks. They used the data of three landmark behavioural object vision studies in rats. Interestingly, for all three studies, they found that the earlier layers could reproduce or exceed the performance of rats, suggesting that the level of visual processing required to solve the tasks was a far cry from the level of processing thought to support primate object recognition. In a next step, Vinken and Op de Beeck compared neural data with the DNN data^17^. Here, they found that the neural representations in the most lateral brain area, i.e. the highest in the visual hierarchy, shows similar representations as for the mid-layers in the DNNs. Vinken and Op de Beeck^17^ thus argue that there is evidence for up to mid-level complexity of object vision in the rodent visual system, which is different and less complex than the primate visual system.

In the current study, we performed a similar comparison between our rat behavioural data and deep neural networks to further investigate the role of contrast features in rat vision. We expect that the earlier layers of a neural network could underlie the importance of contrast features in rat vision.

## Results

### Face categorization training

**Figure 3:**
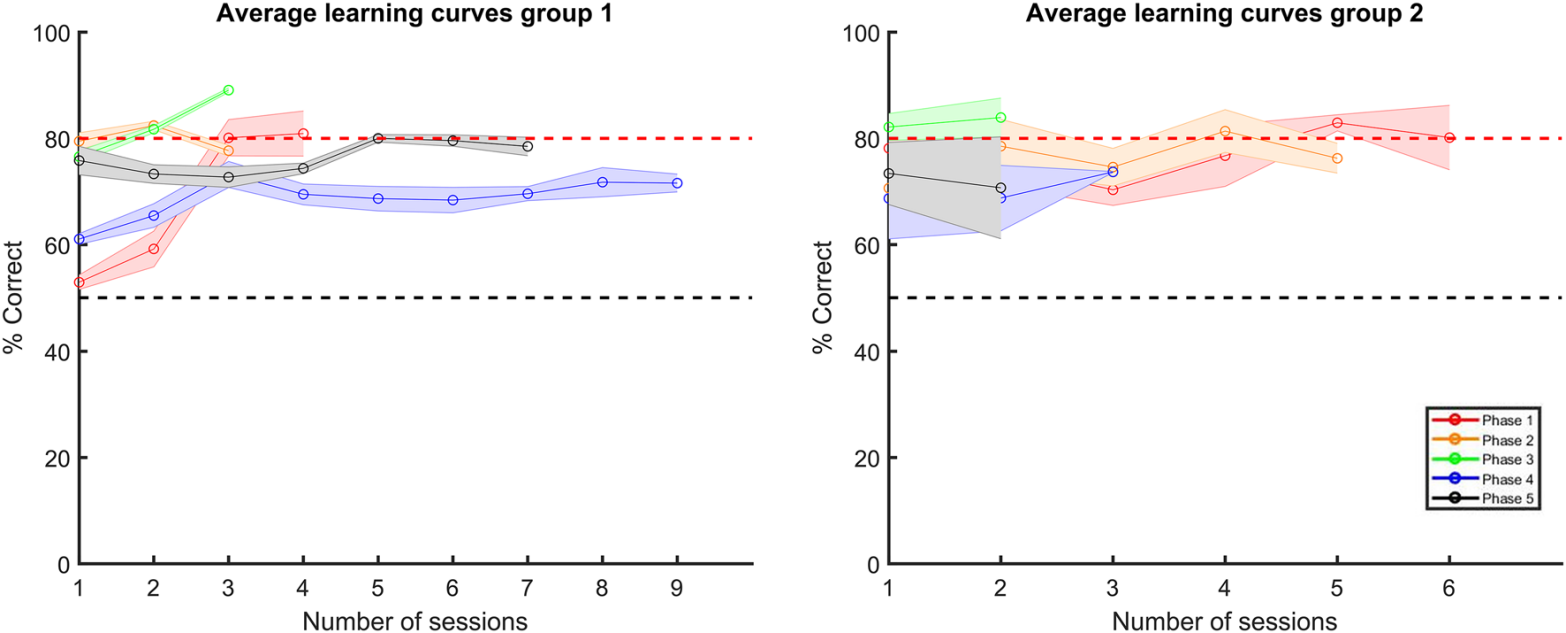
Learning curves of both groups of animals, averaged across animals. The left graph shows the learning curves of the animals that received the training stimuli in the order as presented in Fig. 8a, which were 6 rats in total, whereas in the right graph, the animals, 4 in total, received the stimuli in reversed order. The red dashed line represents the 80% performance threshold, whereas the black dashed line represents chance level. The shaded error bar corresponds to the standard error.

From the learning curves in Fig. 3 it is clear that the animals were able to learn this face categorization task. This figure shows the learning curves per training phase of only the first round, for the pooled responses per group. At each phase, a new training pair was introduced, ranging from one pair in Phase 1 to five pairs in Phase 5. We chose to not include the data of the second round in this Figure, as the animals were not naïve at the start of the second round, and thus their performances were higher to begin with. We display the data of the two groups separately, because they received the stimuli in reversed order. All performances in the first session of the different phases are significantly above chance level for the first group, except for the first phase (binomial test on the pooled response of all included animals, p = 0.34, 95% CI [0.47; 0.60]). Interestingly, the first group had more difficulties with the fourth phase, as can be seen by the larger number of sessions needed for this phase. The second group showed the learning curves as we expected them, and that were similar as in^14^: more sessions for the first two phases, and fewer sessions for the later phases. The second group of animals received the training stimuli in reversed order, as to exclude any order effects of the stimuli. We compared the training performance of both groups, and found no significant difference between these groups (two sample t-test on session performances, t(8) = − 0.47, p = 0.65).

### Generalization

**Figure 4:**
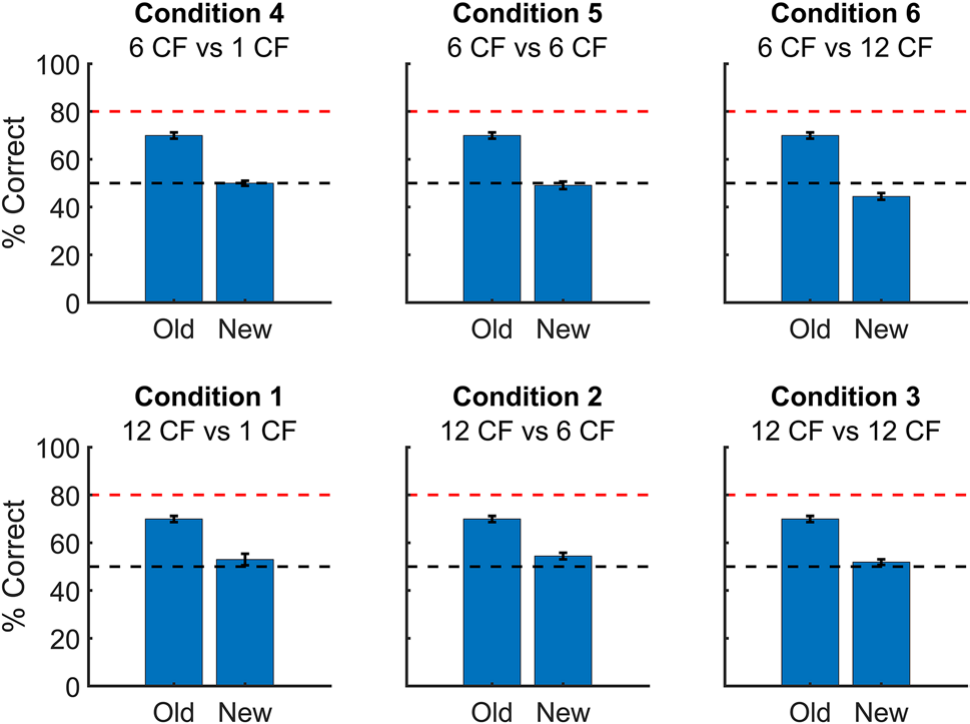
Comparison of the performance of all rats on the new versus the old stimuli, per condition. The error bars indicate the standard error across rats. The red dashed line represents the 80% performance threshold and the black dashed line indicates chance level. The Condition titles refer to the conditions as described in Table 1.

To test the six generalization conditions (see table in Fig. 2), we presented the animals with 10 new targets and 15 new distractors in two rounds of testing protocols. In all our generalization analyses, we combined the data of both rounds as we found no significant difference between the two testing rounds (two sample t-t-test on condition performances, t(14) = 1.97, p = 0.07, with slightly lower performances in the second round). For each condition, we compared the average performance of all rats on the old versus the new stimuli for both rounds together (see Fig. 4). Overall, the average performance on the new stimuli was very low, with an overall performance across all six conditions at 50.45%. This generalization performance was not significantly higher than chance; p = 0.49, 95% CI [0.49, 0.51]) indicating that, overall, the animals do not generalize to new faces when performance is averaged across all 6 conditions. However, the contrast feature hypothesis predicts differences in generalization performance across the six conditions (see table in Fig. 2), to such a degree that we do not expect good generalization performance in several conditions where the number of useful features is not higher or even lower in the target compared to the distractor condition.

The contrast feature hypothesis predicts differences in generalization performance across the six conditions (see table in Fig. 2). The accuracy on the new stimuli for the “12 vs 1 CF” and “12 vs 6 CF” condition was significantly higher than chance level (binomial test on the pooled response of all rats for 12 vs 1 CF: p < 0.05; 95% CI [0.50,0.56]; fir 12 vs 6 CF: p < 0.001; 95% CI [0.52,0.57]), whereas for the 6 vs 12 CF condition the accuracy was significantly lower than chance level (p < 0.0001; 95% CI [0.42, 0.47]). For the remaining three conditions (12 vs 12 CF, 6 vs 1 CF and 6 vs 6 CF), the animals performed at chance level. These results are to a certain extent in line with our hypotheses, suggesting evidence for contrast features in the generalization ability of the animals in this task. However, overall, the accuracy was much lower than expected, especially for conditions 1 and 2. If the animals would have solved the task during training only by means of the aforementioned contrast features, then generalization performance in these 2 conditions should be closer to the accuracy for the training stimuli. Still, the pattern of generalization shows an effect of contrast features, with above chance performance in the condition with the largest difference in contrast features in the typical direction (“12 vs 1 CF”) and performance below chance in the condition with a difference in contrast features in the opposite direction (“6 vs 12 CF”).

**Figure 5:**
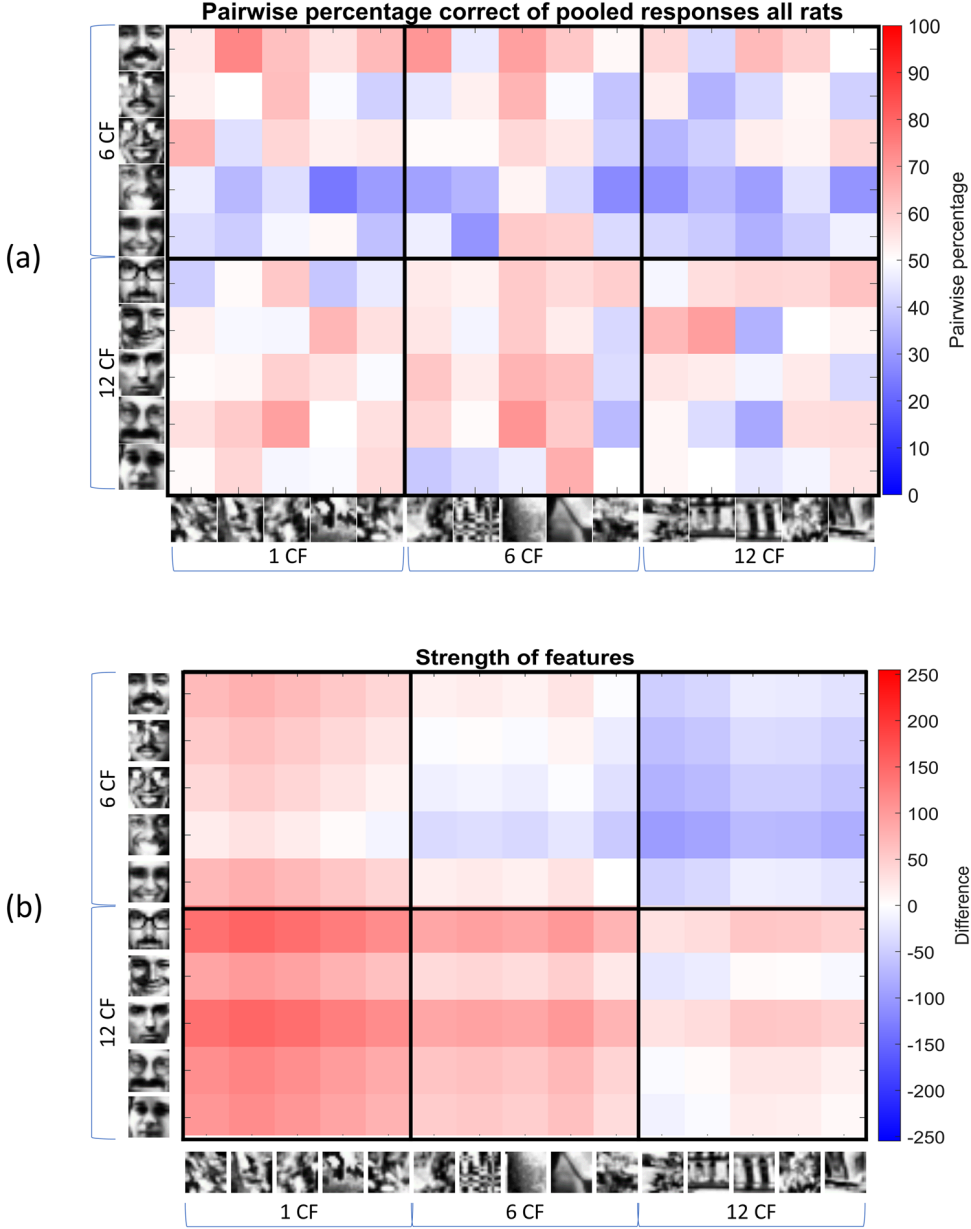
(**a**) Pairwise percentage correct matrix of the pooled response of all rats. The more red a cell is, the higher the performance above chance. Below chance values are indicated in blue. (**b**) The feature strength model. This model indicates how strong each feature is in each stimulus pair. The more red a cell is, the higher the average feature strength of the target, and thus the stronger the features are in the target. In both matrices, the black outlined clusters indicate the conditions as discussed in Figs. 2 and 4.

To further investigate the generalization step, we constructed a pairwise performance matrix, where each cell indicates the average performance across all rats on that specific stimulus pair (Fig. 5a). The more red a cell is, the more above chance the performance on that stimulus pair, and thus the easier this stimulus pair was for the animals on average. Below chance performance is indicated in blue. To test the reliability of this matrix, we calculated the correlation of the pairwise matrix between round 1 and round 2, resulting in a correlation of 0.37. By applying the Spearman-Brown correction^18^ to get the reliability of the full dataset, we obtain a full-set reliability correlation of 0.54.

Ideally, if there would be evidence strongly supporting the role of the number of correct contrast features, we would see a clustering in this matrix where each cluster (or bigger square) would correspond to one of our six conditions (see table in Fig. 2). This clustering is not very clear in the empirical data (Fig. 5a). Still, there are some differences. For example, the cluster or square in the left bottom, corresponding to condition 1, shows more red cells compared to the cluster on the upper right, corresponding to condition 6.

Other interesting findings that can be seen in this pairwise percentage matrix are the within-condition differences between the targets (rows). For example, target 4 seems to be a difficult target, as the average performance on all pairs that includes this target was rather low. Target 1, on the other hand, is even slightly better than targets 6–10. This big difference in performances between target 1 and target 4 is interesting, as they belong to the same category of correct number of features (6 CF).

To further investigate these within-condition differences, we computed a more refined contrast feature model (see Methods) that takes into account the amount of contrast for each feature (“feature strength”) and not only the sign of the feature (is it in the correct or wrong direction). We compared our pairwise matrix with this “feature strength” model (Fig. 5b). The more red a cell is in this matrix, the higher the average feature strength of the target is, and thus the stronger the features in the target (and vice versa for blue cells). This can be either in the positive way or in the negative way. Again, the within-condition difference between target 1 and target 4 becomes apparent. Even though both targets belong to the same condition, i.e. 6 correct features, the strength of their features differ a lot. If we correlate the values in the behavioural pairwise matrix (Fig. 5a) with the prediction of the feature strength model (Fig. 5b), we get a (Pearson) correlation of 0.37 (p < 0.0001) suggesting that this model partially explains the behavioural results, and thus providing evidence in favour of contrast features. To investigate whether this correlation is significant, we performed a permutation, similar as in^14^. We permuted the original pairwise percentage matrix (Fig. 5a) 1000 times, and correlated the values in each permuted matrix with the prediction from the feature strength model (Fig. 5b). This results in a null distribution centred around zero (μ = 0.0038, σ = 0.08). To determine significance, we investigated how many of those 1000 correlations are larger than, or equal to the empirically observed correlation of 0.37, and divided this number by the number of permutations (1000). We obtain a p-value of < 0.001, indicating that the correlation between the pairwise percentage matrix and the feature strength model is indeed significant. Keeping the higher Spearman-Brown corrected reliability of 0.54 in mind, there might still be meaningful variability in the behavioural data that is not explained by the contrast feature model. Yet it is clear that large part of the meaningful variability is captured with a model that takes into account contrast features and their strength.

### Deep neural networks

**Figure 6:**
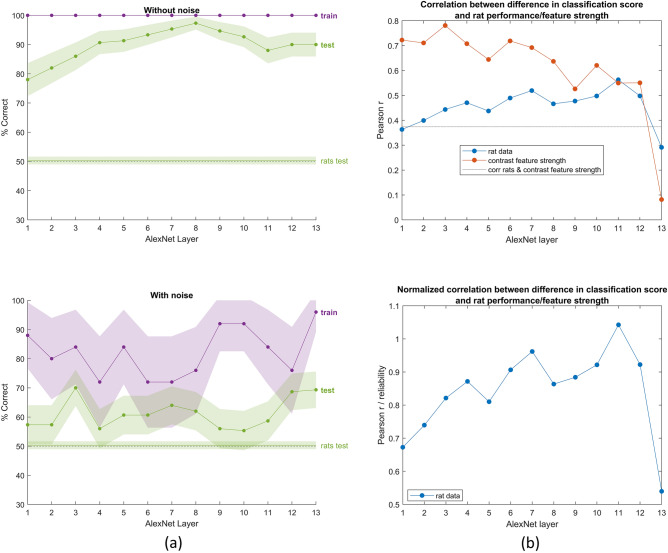
(**a**) The performance of the network after training on our training stimuli, without (top panel) and with noise (bottom panel) added to the data. The purple line indicates the training performance, the green line with dots indicates the test performance of the neural network. The green horizontal line indicates the average generalization performance of the animals, whereas the dashed horizontal black line indicates chance level. The x axis on both graphs indicates the block of layer: layers 1–13 on the x-axis correspond to convolutional layer 1, normalization layer 1, pool layer 1, convolutional layer 2, normalization layer 2, pool layer 2, convolutional layer 3, convolutional layer 4, convolutional layer 5, pool layer 5, fully connected layer 6, fully connected layer 7 and fully connected layer 8, respectively (see our Supplemental Table S1 for an overview of the layer blocks that we use). The shaded error bars correspond to 95% confidence intervals calculated using Jackknife standard error estimates, as done previously in^17^. (**b**) Correlation of the classification score for single target/distractor pairs between single DNN layers and either the rat performance (blue) or the contrast feature strength model (red). The dotted horizontal line indicates the correlation between the rats performance and the contrast feature strength (0.37). The lower panel in (**b**) shows the same data for the correlation with the rat data, but normalized on the reliability of the rat data after the Spearman-Brown correction. The same layer naming convention on the x axis as in (**a**) is used.

A final step was to use AlexNet, train a linear classifier on the unit responses of single layers on to classify the 10 training stimuli and compare the generalization of this DNN classifier to the test stimuli with the generalization behaviour of the rats. Figure 6a (upper panel) shows the performance of the network after training on our training stimuli. The network had no problems to achieve high performances in the testing phase, even not with relatively low layers in the network. This performance can be considered as unrealistic because the network makes zero errors in the training, which contrasts with the relative high lapse rate in the rats. For this reason, we added noise to the network so that the network performance averaged across all training stimuli equated rat performance during training. When adding noise to the model, our findings change substantially (Fig. 6a, lower panel). Overall, the generalization performance of the model is quite low, with a maximum of about 65%. For some layers, the performance even drops to almost 50%, such as for layer blocks 1, 4 and 11 (see Supplemental Table S1 for how we divided the layers into blocks). Still, it is higher than the generalization performance of the rats.

Next, we investigated how the variation in generalization performance across image pairs correlates between the DNN classifier and rat performance. Figure 6b (upper panel) displays per DNN layer the correlation of the target-distractor difference in classification scores (i.e., difference in signed distance to hyperplane) for single target-distractor pairs, with the corresponding rat performance values (blue line). Higher layers show more similarity to rat performance in terms of the across-pair variation, at least up to layer 11 (first fully connected layer). The correlations reach the highest values that we can expect given the reliability of the behavioural data, which is visualized in Fig. 6b (lower panel) by dividing the correlations from Fig. 6b (upper panel) by the reliability of the data after correcting through the Spearman-Brown formula.

Finally, we investigated to what extent the DNN classifier makes the same prediction as the contrast feature strength model. Or, said otherwise, to what extent contrast features are captured by DNNs. For this analysis, we calculated for each image pair how confident the classifier was of its decision in a similar manner as we did for the rat data, i.e., captured by the (signed) distance to its decision hyperplane (see Methods). The red curve in Fig. 6b (upper panel) reveals a high correlation, most prominently for the lower DNN layers. This figure suggests the existence of a small interaction between the hierarchical level in DNNs and whether we correlate DNN predictions with the contrast feature strength model or with actual rat behaviour (Fig. 6b): earlier layers show more correspondence to the feature strength model (Fig. 5b), whereas higher layers show more similarities to the rat behavioural performances (Fig. 5a). Taking the interaction as a whole, this means that as the network relies less on contrast features, correlation with rat performance goes up. Given that the correlations with the feature strength model are high, these data do not contradict that rats and DNNs use contrast features in general. Nevertheless, the aforementioned interaction indicates that in addition to contrast features, there are other image features that influence rat performance. These additional features seem to be captured by later layers of the tested DNN, whose performance was correlated perfectly (cfr. the normalized correlations in Fig. 6b, lower panel) with behavioural performance.

## Discussion

In the current study, we wanted to investigate the role of contrast features in rodent vision. We trained the animals in a face categorization task and tested how well they could generalize to new exemplar stimuli, using protocols similar to Schnell and colleagues^14^ but now using stimuli that were previously used in computer vision and in primate research. The stimuli were chosen based on their contrast features, i.e. features that capture pairwise contrast relationships across two facial regions. We combined our hypotheses in a contrast model (Fig. 2), and compared this model to the performances of our animals. To have a more detailed look at the data, we created a more refined contrast feature strength model (Fig. 5) which takes the weights of the contrast features into account. Finally, we compared the contrast model and our feature strength model to the layer performances of a deep neural network (DNN). Together our findings provide evidence in favour of contrast features, yet there are also other features that play an important role in the performance of our animals.

During training, the first group of animals received the stimuli in the order as presented in Fig. 8a, and they showed some difficulties after introducing the fourth and fifth stimulus pair. We ensured high pixel dissimilarity within the training stimulus set, and so this would not explain why the first group of animals needed more sessions with the fourth and fifth stimulus pair (Supplemental Fig. S2).

After successfully training the animals in the categorization task, the animals continued with the generalization phase. Here, they were presented with 10 new targets and 15 new distractors to test our hypotheses (table in Fig. 2). When looking at the rat performances on these testing stimuli (Fig. 4), they showed an overall low generalization performance. Regardless of the low generalization performance, the performance pattern across testing conditions was moderately in line with our hypotheses, with above-chance performance in several of the conditions where contrast features could support correct face categorization and below-chance performance in the condition where contrast features were going against correct face categorization. This supports the claim that contrast features influence task performance. Still, the observation that generalization performance was low overall indicates that the animals used more information during training than just the contrast features. One possible explanation is that the animals only learned these specific five training pairs, similar as if a neural network would overfit the training data. The animals would then be unable to perform well for unseen stimuli, as is the case in our behavioural results. Another explanation is that the animals might focus on pixel similarity to perform this task. Because we ensured high pixel dissimilarity between the training and testing stimuli, a focus on pixel similarity would result in a low generalization performance. A final possible explanation is the low number of training pairs. Perhaps the low number of training stimuli (5 targets and 5 distractors) is not enough to capture the essence of contrast features. Still, we do find evidence for the use of contrast features in our behavioural data, so their importance was picked up by the animals to at least some degree.

Using only the generalization performance of the animals in the six conditions did not provide a very detailed picture of how the animals solve this task. We therefore took a more detailed look at the stimuli and performances by calculating the pairwise percentage matrix for each target-distractor pair. We found some interesting within-condition differences suggesting that the number of correct features might be too simple as a metric, given that features can differ in how big the differences are. This led us to create the feature strength model. Here, we gave weights to each correct feature based on how big the contrast is for that feature. This model correlates well with the variation in behavioural performance across stimulus pairs, suggesting again that the animals, to some extent, use contrast features. To further investigate what might predict the performance of our animals, we also compared their performances, i.e. their pairwise performance during generalization, with the pairwise pixel dissimilarity of these stimuli. We found a very low correlation (correlation = 0.06), excluding pixel dissimilarity as an important factor. This finding is probably related to how we used pixel dissimilarity to decide which stimuli to use as training and test stimuli, because if pixel dissimilarity would have allowed a clustering of faces versus non-faces, then it would very likely have played a role. This leaves us with the question what the driving factor is behind the performance of the animals.

The question whether rats use contrast strategies has been discussed in other studies as well. Vinken, Vermaercke & Beeck^19^ for example discussed the possibility of rats comparing the luminance of parts of the stimuli. Another possibility, as suggested by^19^, is that the animals use contrast strategies, but the way they do it is more complex because they might combine multiple contrast cues, which is exactly what we are reporting in the current study. The use of contrast features is also consistent with the earlier findings of Vermaercke and Op de Beeck^10^, who suggested that rats adopt flexible contrast strategies. Vermaercke and Op de Beeck^10^ used a local occlusion paradigm, the Bubble paradigm, to get insight into the visual templates of rats performing a visual discrimination task. They suggested that rats used a strategy that can be verbalized as “If the bottom of the screen is filled with grey, then look at the top middle of the screen and avoid whichever is brighter.” Such a strategy also refers to contrast features.

To get a more fine-grained computational understanding of the strategies the animals use, we compared the predictions of a deep neural network with rat behaviour and with the contrast feature models. We used AlexNet and tested how well a classifier trained on specific layers of the network could generalize to the test stimuli, after we trained it on the training stimuli. We found that overall the classifier could generalize to our testing stimuli, even with the lower layers. When comparing the variation in generalization prediction across image pairs, we found that the lower DNN layers showed a similarity with the feature strength model. Although contrast features can influence neural responses even in higher areas of the primate visual system^7^, their computation is relatively simple and was originally proposed in the context of simple computer vision models. From that perspective it is not very surprising that such features can be captured by lower hierarchical levels in the visual system.

It is more surprising to find that overall the higher DNN layers were even better at explaining the variation in rat performance. It is quite exceptional to find such evidence for higher-level processing strategies when using DNNs as a benchmark. Earlier suggestions of higher-level processing did not always survive this test. Djurdjevic et al.^20^ investigated how well rats could discriminate a target object from 11 distractor objects. They found that the animals are capable of more complex, advanced shape processing. Another study that provides evidence for high-level visual processing in rats is the study of Zoccolan et al.^9^. They trained rats in an invariant object recognition task where the objects differed in size, view and lighting. This forced the animals to adapt their visual strategy in this task, and^9^ suggested that these animals were able to perform well in this complex task. However, the later computational tests by^17^ suggested that the behavioural data obtained by^9,20^ could be explained to a large extent mostly by representations in lower convolutional layers, most so in the case of Djurdjevic et al.^20^.

The DNN modelling does not pinpoint the nature of these higher-level strategies. Coming back to the distinction in the Introduction between strategies in terms of shape features or contrast features, there can also be hierarchy of complexity in terms of contrast features. Other work has shown that standard DNNs like the pre-trained AlexNet that we used tend to process images in terms of texture rather than shape^21^. The observation that later layers in such a texture-biased network can fully (Fig. 6b, lower panel) capture the performance of rats in the current paradigm, strongly suggests that we do not need to invoke shape-based processing to explain the behaviour of rats in our face categorization experiment. Overall, our findings are in line with the importance of a hierarchy of contrast features for explaining rat behaviour in visual object recognition tasks.

The interpretation of our findings and their relevance for cross-species comparisons between rats and primates should be considered against the background of other known differences between these species. Up until 20 years ago, monkeys were the main animal model of choice in vision research. With the rise of new neurotechnology and genetic rodent models, rodents became more popular. Nevertheless, the question remains to what extent the rodent vision system is comparable to the primate and human vision system. Despite the differences in neuroanatomy, rodents, and more specifically rats, have shown to be able to perform well in rather complex visual object recognition tasks (for a review, see^8^). This suggests that for at least some questions, rodents could be used as an alternative model to study higher-level vision (^8^). To further test this, past studies have compared the performance of rats and humans in complex visual tasks. In some studies, rats perform worse than humans^12^. In this study, rats and humans were presented with a linear and non-linear object discrimination task. Rats were unable to learn the nonlinear task, whereas humans had no difficulty of either task. Likewise,^22^ even found that rats were not able to learn a relatively easy shape discrimination task (square versus rectangle) if it required the extraction of a shape feature like aspect ratio. Other studies, however, found the opposite results, i.e. they describe tasks where rats perform surprisingly well. Vermaercke et al.^11^ for example found that rats reached higher performances in an information-integration categorization task than humans, at least when taking the performance on one-dimensional tasks as a reference. While these previous studies compared rats to humans, a further question is where other primate species fall, in particular primate species that stand relatively far away from humans. Kell et al.^23^ performed a simple visual recognition task on marmosets and compared their performance to that of rats. Interestingly, marmosets remarkably outperformed rats. Because all of these studies are a bit scattered, comparing few species and specific tasks that are often optimized for a specific species, it will be a promising line of research to design a complex visual recognition task that allows to compare the full scope of visual abilities of a larger number of species.

An important point when designing further tasks, is the ecological validity given the natural behavior and habitat of a species. Obviously, the task that we used, visual face categorization, is not an ecologically valid task for rats. This lack of ecological validity could explain the low generalization performance of the animals. Face detection is an artificial task for rats, and they had to learn this task from scratch. We should not take the limited performance of rats in this task as a valid indication of their visual capabilities, which might be more sophisticated in lab tasks modeled after natural behavior such as navigation and prey/predator situations^24–27^. However, our goal in the current manuscript is to provide a direct demonstration that rats use a strategy in terms of contrast features. Given that for humans this hypothesis has been most clearly formulated in the case of human face detection, it made sense to also test this domain in rats. The current study, in which contrast features were explicitly manipulated, provides direct evidence for the use of contrast features. This confirms the suggestions from previous studies in which the inferred strategies of rats seemed to align well with an interpretation in terms of contrast features, in domains varying from simple shape categorization^10^ and video-based rat/nonrat categorization^19^. It remains to be tested whether this contrast feature hypothesis will also hold in the wide variety of tasks that rats perform naturally.

In summary, we tested a face categorization task in rats, using stimuli that are parameterised in terms of contrast features to investigate whether rats use these low-level visual features, given the knowledge that humans and monkeys use them in face detection. We found evidence that the animals use contrast features as at least part of their strategy. We discussed our results in the light of ecological validity as well as differences between species, such as rats versus humans and rats versus monkeys.

## Methods

### Animals

A total of ten male outbred Long Evans rats (Janvier Labs, Le Genest-Saint-Isle, France) started this behavioural study. Two of the ten animals were pilot animals from a previous electrophysiological experiment and were 35 and 48 weeks old at the start of the current experiment. These two animals were housed individually because of their headposts. The remaining eight animals were about 12 weeks old at the start of training and were housed in groups of four per cage. Each cage was enriched with a plastic toy (Bio-Serv, Flemington, NJ) and paper cage enrichment. Halfway through the experiment, one pilot animal was excluded because it did not perform high enough on the training stimuli during the test protocols, suggesting it forgot the learned behaviour. During the second part of the experiment, one animal was excluded because of health issues. Thus, a total of eight animals completed the experiment. During training, rats were food restricted to maintain a body weight of about 85% of their original body weight. They received water ad libitum. All experiments and procedures involving living animals were approved by the Ethical Committee of the University of Leuven and were in accordance with the European Commission Directive of September 22, 2010 (2010/63/EU). We have reported the study in accordance with the ARRIVE guidelines.

### Setup

The setup was identical to the one used by Schnell and colleagues^14^. A short description will follow here. All rats were tested in four automated touch-screen rat-testing chambers (Campden Instruments, Ltd., Leicester, UK) with ABET II controller software (Lafayette Instrument’s versatile ABET II v2.18 and Whisker’s Control system (WhiskerServer) v4.5.0). On one side of the chamber, a reward tray was installed in which sugar pellets (45-mg sucrose pellets, TestDiet, St. Louis, MO) could be delivered. On the other side of the chamber, an infrared touchscreen monitor was installed which was covered with a black Perspex mask containing two square response windows (10.0 × 10.0 cm). A shelf (5.4 cm wide) was installed onto the mask (16.5 cm above the floor) to force the animals to view the stimuli within their central visual fields.

### Stimuli

All stimuli were retrieved from Ohayon et al.^7^ and included a set of 207 faces and 204 non-faces, measuring 100 × 100 pixels. The face stimuli were front-view human faces in grey-scale. The non-face stimuli were random samples from natural images that do not include human faces^7^. Based on two stimulus variables, we chose 10 training stimuli consisting of 5 faces (targets) and 5 non-faces (distractors) and 25 testing stimuli, consisting of 10 faces and 15 non-faces.

**Figure 7:**
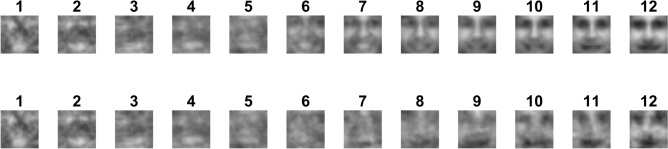
Results of the template similarity measurement. The top row indicates the average image of all faces belonging to a certain number of correct features. The number of correct features is indicated by the numbers on top of each image, i.e., each column corresponds to one correct feature category. The bottom row shows the average image of all distractors belonging to such a category. Interestingly, the average image of all distractors with 12 correct features resembles a face, whereas the average image of all distractors with only 1 correct feature resembles an inverted face.

The first stimulus variable that was used to indicate the targets and distractors is *template similarity*. This variable indicates how similar a stimulus is to the face template created by Sinha^5^. We started by calculating the template similarity used by Ohayon and colleagues^7^, following the steps that can be found in their Fig. 6. First, we copied their template (their Fig. 6A, see our Fig. 1) and overlaid each stimulus with this template. We then calculated the number of correct features in an identical manner as they have. The twelve conditions in Fig. 1 were tested, which resulted in a certain number of “correct contrast features” per stimulus, i.e., how many of these twelve conditions are met, and thus quantifying how face-like a stimulus is. A next step was to sort all stimuli into 12 categories depending on their number of correct contrast features (see their Fig. 6B). Figure 7 shows the average image of all stimuli belonging to each category, based on the template similarity.

The second stimulus variable was *pixel dissimilarity*, which we used to avoid that the selected images in each category would be very similar in all respects. *Pixel dissimilarity* was calculated within each category of the template similarity. To calculate the pixel dissimilarity of a stimulus pair, we followed the method of^28^. For each pair of stimuli, we first computed the difference in each pixel, squared this difference and summed it across all pixels. We then took the square root of this sum and normalized the resulting number by the square root of the number of pixels. This results in a difference or dissimilarity measure^28^. We computed the pixel dissimilarity for each pair of stimuli within a category of the template similarity, that is, stimuli that have the same number of correct contrast features. This resulted in one dissimilarity matrix for each number of correct features. Ideally, only stimuli with a high dissimilarity to each other were chosen, to avoid a confounding between template similarity and pixel dissimilarity.

**Figure 8:**
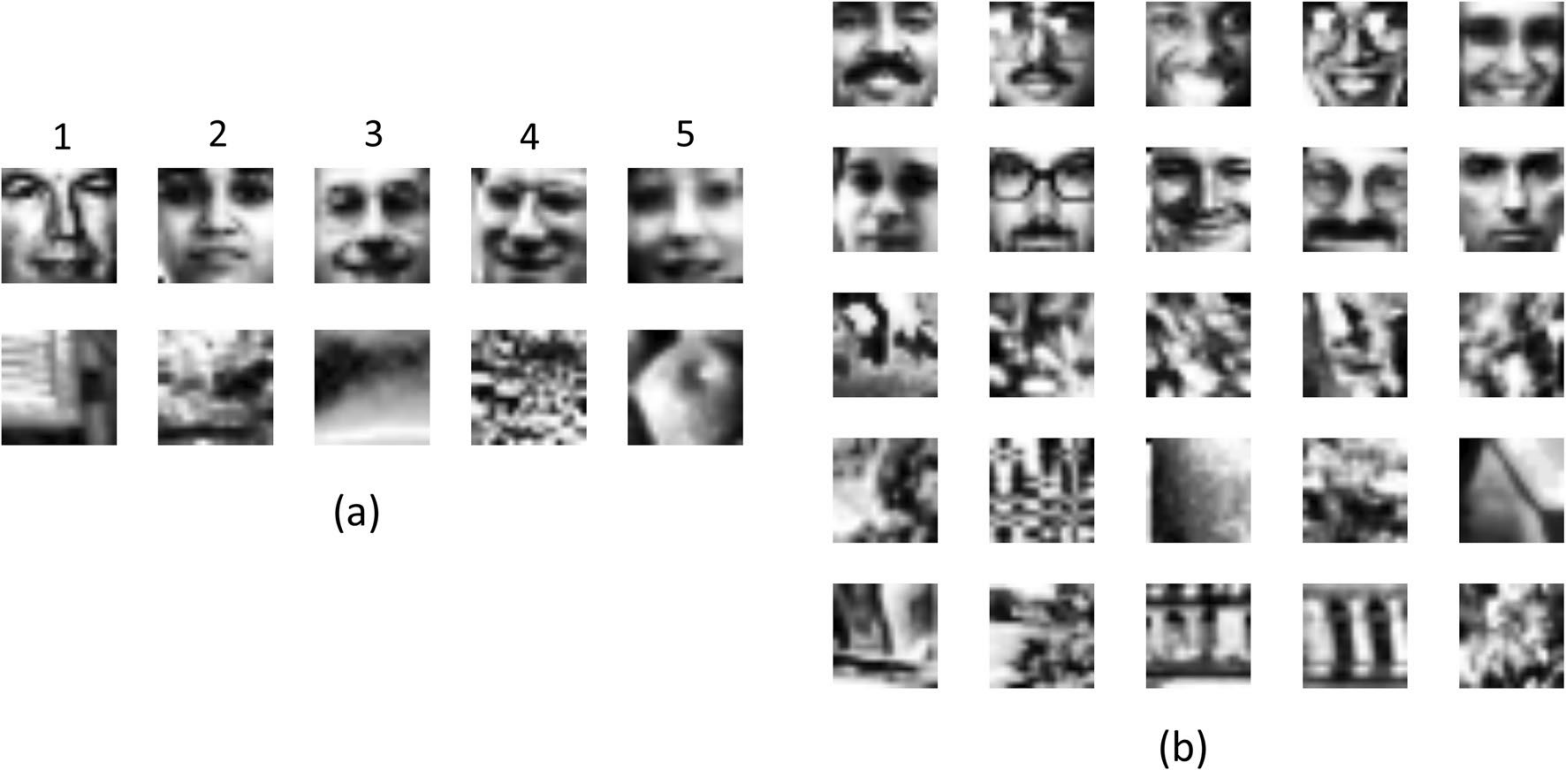
(**a**) The training stimulus set consists of five targets and five distractors. One group of animals received the stimulus pairs in the shown order, whereas the second group received them in reversed order to ensure that there is no effect of stimulus order. (**b**) All testing stimuli. The upper two rows visualize the testing targets (i.e., faces) with a different number of correct contrast features. The top five targets have 6 correct contrast features, whereas the bottom row of faces includes targets with 12 correct contrast features. The lower three rows visualize the testing distractors (i.e., non-faces), again with a different number of correct features (1, 6 and 12) corresponding to our hypotheses.

Taking these two stimulus variables into account, a training stimulus set of 5 targets and 5 distractors was constructed, as well as a testing stimulus set of 10 targets and 15 distractors (Fig. 8). The training targets each had 11 or 12 correct contrast features, whereas the distractors each had 6 correct contrast features. The testing stimuli corresponded to the 6 conditions that were mentioned in our hypotheses, and thus we chose testing targets with 6 or 12 correct contrast features, and testing distractors with 1, 5 or 6, or 11 or 12 correct contrast features. For the training part of this experiment, half of the animals were presented with the order shown in Fig. 8a, whereas the other half received the reversed order to examine a possible effect of stimulus order. The dissimilarity matrix of this set of stimuli (see Supplemental Fig. S1) shows that this is indeed a good stimulus set to use, as there were many dissimilar stimuli as suggested by the many red cells in the matrix. Important to note is the high dissimilarity within the training set as evidenced by the many red cells within the training square, but also the high dissimilarity of the test set relative to the training set, shown by the red cells of the testing stimuli compared to the training stimuli. Pixelwise dissimilarity in the chosen set does not show an obvious clustering in terms of face versus nonface or number of correct features.

In a further analysis, we computed a more refined contrast feature model that takes into account the amount of contrast for each feature (“feature strength”) and not only the sign of the feature (it is in the correct or wrong direction). In other words, rather than counting whether or not a condition (one of the twelve features) was met, we calculated the difference in pixel values for each condition in the correct (positive values) or incorrect (negative values) direction. If, for example, the brightness of the forehead is higher than the brightness of the left eye, which is an expected condition for a face, then we wanted to know how large the difference in brightness is. We calculated these differences, which we will call “feature strengths”, for each stimulus and for each condition. This resulted in twelve values for each stimulus, corresponding to the strength of each feature. To get the value of each cell of the feature strength matrix, we calculated the average of these twelve values for each target and each distractor. We then subtracted the distractor average from the target average because we also look at this difference in our hypotheses (target–distractor). The higher this value, the higher the average feature strength of target is and thus the stronger the features are in the target relative to the distractor.

### Protocols

The design of the experiment is very similar to the design of Schnell et al.^14^. A short description will follow here. Animals started with a standardized shaping part to get accustomed to the touchscreen setup (see^14^ for details). This part consisted of five phases, starting with a simple habituation where the animals are placed in the touchscreen setup without any stimuli presented to the screen. In each phase, the animals learned one extra thing, for example in the first phase they learned that touching the screen results in a (food) reward and in a later phase they learned that a trial can be initiated by placing their nose in the food area.

Identical as in^14^, rats started with the face-versus-nonface discrimination training once they successfully finished the shaping procedure. The animals performed a single session each day and each session lasted either 100 trials or 60 min. In between two trials, we added an intertrial interval of 20 s. During training, correction trials were presented after an incorrect response to reduce the chance that animals would develop a response bias to one of the two screens. We used a training performance threshold of 80%, meaning the animals had to perform at or above 80% correct or higher for two consecutive sessions before proceeding to the next training phase. For three animals however, reaching 80% correct or higher seemed difficult, and thus the criterium was relaxed in an identical manner as in^14,29^. In those animals, the threshold was lowered such that they had to perform at 75% correct or higher during four consecutive sessions. Additionally, their performance correct during the last sessions had to be at or above 75%.

The training procedure consisted of five phases. In the first phase, the animals were shown the first face-nonface pair (Fig. 8a). Touching the screen with the face, i.e. the target, was rewarded in all trials. After reaching the performance criteria in this first phase, rats continued with the second training phase, where the second target (face) and distractor (non-face) were added. All possible combinations of faces versus non-faces were randomly presented. In the third, fourth and fifth training phase, the third, fourth and fifth target and distractor were added to the stimulus set. Rats were trained until they could discriminate the five faces in Fig. 8a from the five non-faces in Fig. 8a at the performance threshold described earlier.

Once the animals were fully trained, i.e., they achieved the performance threshold in the fifth training phase, they continued with the testing protocols. We created a total of three test protocols, each testing a subset of our six hypotheses in Fig. 2. The first, second and third test protocol compared all targets with distractors having 1 or 6, 1 or 12 and 6 or 12 correct contrast features, respectively. In every test protocol, we presented 1/3 old stimuli, i.e. the stimuli used during training, to have a quality check on the performance of the animals. We gave random reward in 80% of the trials with a new stimulus pair, i.e. the stimuli in Fig. 8b, and real reward for old stimulus pairs. We chose a random reward in 80% of the new, i.e. testing, trials, to keep the animals motivated throughout testing. We performed two rounds of testing these three test protocols. For the first round, the testing part consisted of three consecutive blocks, each containing all three test protocols. For the second round, we added a training phase in between each block to ensure high performance on the training stimuli and thus the original face discrimination task. In both rounds, the order of the test protocols was counterbalanced between animals.

### Computational modelling

We investigated the performance of deep convolutional neural networks (DCNNs) when trained and tested with the same stimuli that we used in rats. We used a similar approach as in Vinken and Op de Beeck^17^ to calculate these performances. A short description will follow here. We used the standard AlexNet DCNN architecture. This network is pre-trained on ImageNet to classify images into 1000 object categories and has been taken from the MATLAB 2017b Deep Learning Toolbox. This network is often used as a computational model for visual processing in the primate visual ventral stream^16,30,31^. It consists of convolutional and max pooling layers, followed by three fully connected layers. Additionally, it also includes local response normalization layers. A rectifying linear activation function (ReLU) is added after each convolutional and the first two fully connected layers. We trained a linear support vector machine classifier (the MATLAB function *fitclinear*, with limited-memory BFGS solver and default regularization) on the unit responses (before ReLU) of single layers in this network to our 10 training stimuli (Fig. 8a). For our analyses, we divided the layers of AlexNet into 13 sublayers (Supplemental Table S1).

To examine the accuracy of the model, we calculated the signed distance to hyperplane (classification score) for each target and distractor, with positive values for the target side, and negative values for the distractor side, as done previously in^17^. A trial was considered correct if the difference between the classification score for the target and that for the distractor (target–distractor) was positive; that is, if the model classified the target stimulus as more target-like than the distractor.

## Supplementary Information

Supplementary Information.

## Data Availability

The data has been made publicly available via the Open Science Framework and can be accessed at https://osf.io/52pa3/.
