# Supplementary material for: The importance of contrast features in rat vision
Source: Sci Rep. 2023 Jan 10;13:459. doi: 10.1038/s41598-023-27533-3 (PMC9832064; doi:10.1038/s41598-023-27533-3)
Supplement: Supplementary file 1 — Supplementary Information. [file 41598_2023_27533_MOESM1_ESM.docx]

**Supplemental material**


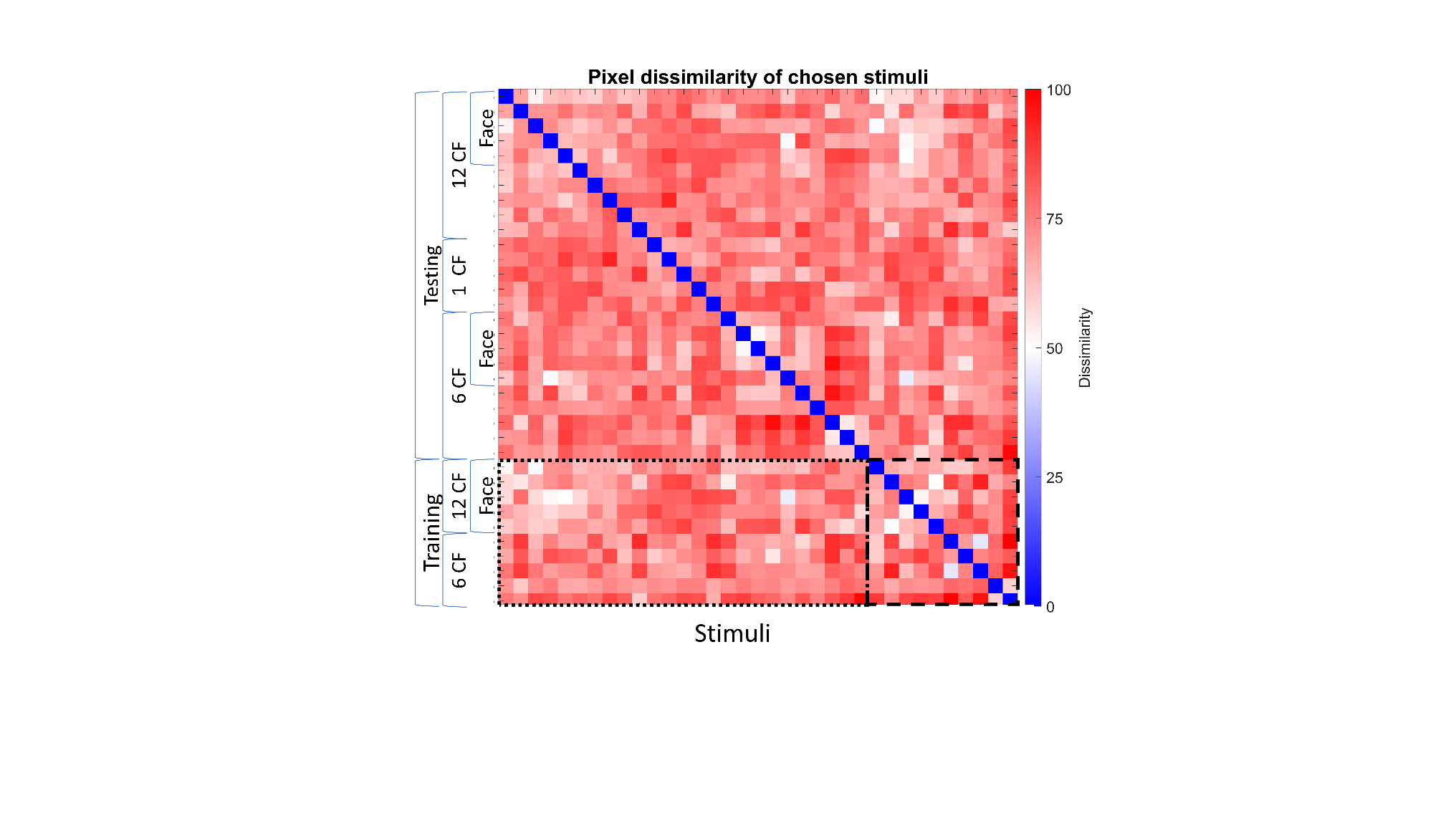


**Supplemental Figure 1** **The pixel dissimilarity matrix of the chosen stimulus set.** The colorbar indicates pixel dissimilarity values, normalized between 0 and 100. The higher this value, the more dissimilar two stimuli are. The black dotted frame indicates the dissimilarity of the testing set relative to the training set, and the black dashed frame highlights the dissimilarity within the training set. As indicated by the red cells, all stimuli pairs have a high dissimilarity from each other


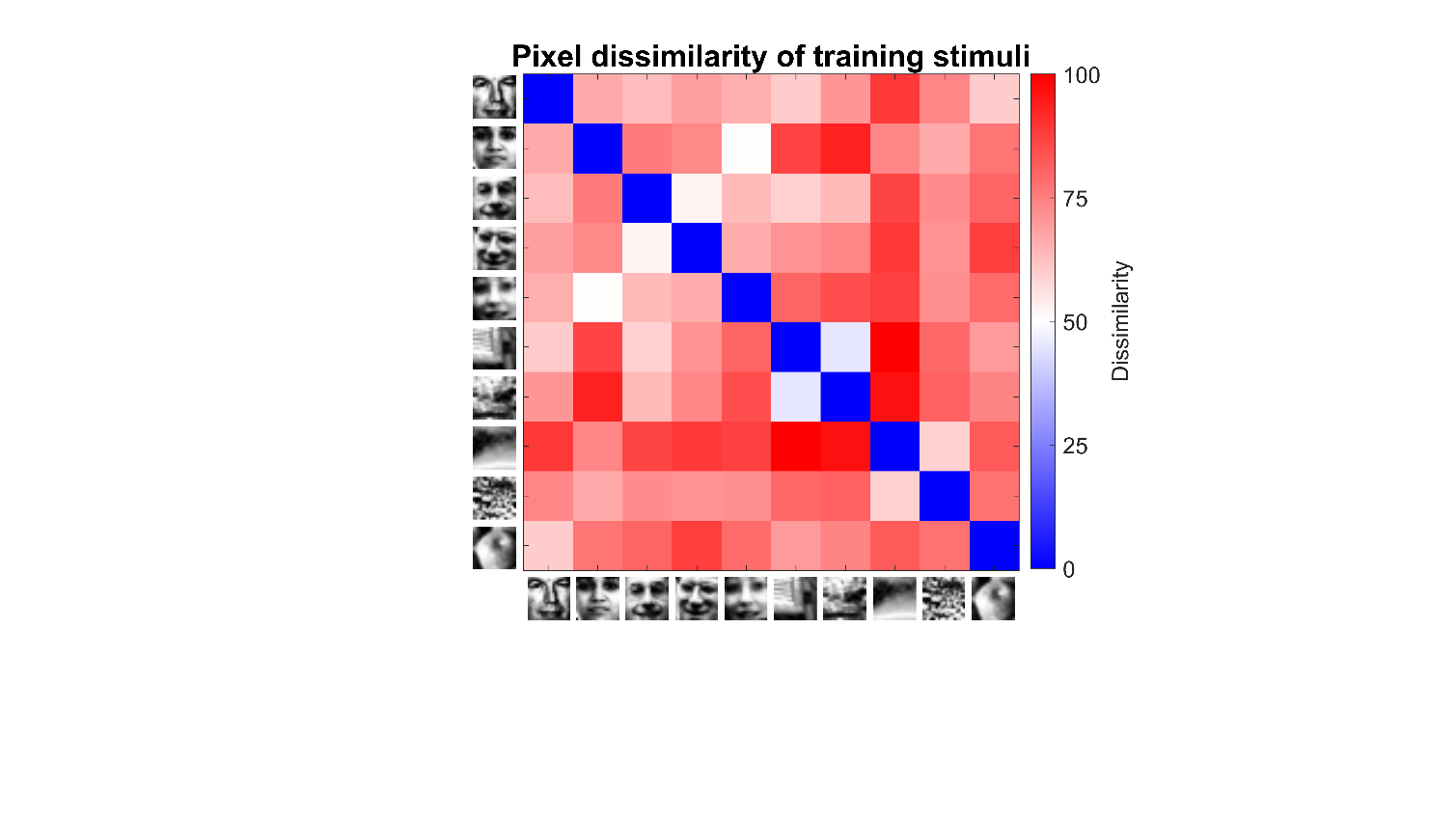


**Supplemental Figure 2 The pixel dissimilarity matrix of the training stimuli.** The colorbar indicates pixel dissimilarity values, normalized between 0 and 100. The higher this value, the more dissimilar two stimuli are.

**Supplemental Table 1** **Division of the AlexNet layers as used in our analyses.** ‘conv’ corresponds to convolutional layer, ‘norm’ indicates the local response normalization layers, ‘pool’ stands for max pooling operation, and ‘fc’ indicate the fully connected layers.

| Layer block | AlexNet layer |
| --- | --- |
| 1 | conv1 |
| 2 | norm1 |
| 3 | Pool1 |
| 4 | conv2 |
| 5 | norm2 |
| 6 | pool2 |
| 7 | conv3 |
| 8 | conv4 |
| 9 | conv5 |
| 10 | pool5 |
| 11 | fc6 |
| 12 | fc7 |
| 13 | fc8 |
